# Chronic Periodontitis as a Risk Factor for Benign Prostatic Hyperplasia: A Cohort Study

**DOI:** 10.3390/jcm14041279

**Published:** 2025-02-14

**Authors:** Mi Jung Kwon, Ho Suk Kang, Hyo Geun Choi, Joo Hee Kim, Dae Myoung Yoo, Na Eun Lee, Kyeong Min Han, Woo Jin Bang

**Affiliations:** 1 Department of Pathology, Hallym University Sacred Heart Hospital, Hallym University College of Medicine, Anyang 14068, Republic of Korea; mulank99@hallym.or.kr; 2 Division of Gastroenterology, Department of Internal Medicine, Hallym University Sacred Heart Hospital, Hallym University College of Medicine, Anyang 14068, Republic of Korea; hskang76@hallym.or.kr; 3 Suseo Seoul E.N.T. Clinic, 10, Bamgogae-ro 1-gil, Gangnam-gu, Seoul 06349, Republic of Korea; mdanalytics@naver.com; 4 Division of Pulmonary, Allergy, and Critical Care Medicine, Department of Medicine, Hallym University Sacred Heart Hospital, Hallym University College of Medicine, Anyang 14068, Republic of Korea; luxjhee@gmail.com; 5 Hallym Data Science Laboratory, Hallym University College of Medicine, Anyang 14068, Republic of Korea; ydm@hallym.ac.kr (D.M.Y.); intriguingly@hallym.ac.kr (N.E.L.); km.han@hallym.ac.kr (K.M.H.); 6 Department of Urology, Hallym University Sacred Heart Hospital, Hallym University College of Medicine, Anyang 14068, Republic of Korea

**Keywords:** periodontitis, benign prostatic hyperplasia, risk factors, epidemiology

## Abstract

**Objective**: The association between periodontitis and benign prostatic hyperplasia (BPH) has been controversial. This study aimed to estimate the association between recurrent periodontitis episodes and the occurrence of BPH in an adult male population in Korea. **Methods**: This study analyzed data from 79,497 matched cases and controls to examine the relationship between periodontitis and BPH, using Korean National Health Insurance Service-Health Screening Cohort data. Conditional logistic regression was used to calculate odds ratios (ORs) with 95% confidence intervals (CIs), adjusting for confounding factors. **Results**: The odds of BPH were significantly higher for participants with periodontitis ≥ 1 within 1 year (OR = 1.34, 95% CI = 1.31–1.37), particularly in low-income individuals (OR = 1.43, 95% CI = 1.38–1.48). Increased periodontitis frequency (≥2 or ≥3 within 1 year) and a CCI score ≥ 2 were associated with progressively higher odds of BPH, indicating that periodontitis may be a significant risk factor for BPH, with variations depending on socioeconomic and health status. **Conclusions**: The occurrence of BPH was higher in participants with a history of recurrent periodontitis episodes, with stronger associations observed in those with low income or multiple comorbidities. Clinicians should be aware of the potential risk of BPH in patients with recurrent periodontitis episodes. This study’s retrospective design, reliance on ICD-10 codes without details on disease severity, and focus on Korean citizens over 40 limit its ability to establish causality and generalizability to other populations and age groups, which should be considered when interpreting the findings.

## 1. Introduction

Chronic periodontitis (CP) is a chronic inflammatory disease of the periodontium, including the gingiva and other supporting structures of teeth. CP is not merely a bacterial infection but is now understood as a result of a dysbiotic subgingival biofilm and dysregulation of the host immune response, which drives the disease process [1]. This concept represents a shift from the traditional view of periodontitis as an infection caused solely by specific pathogenic bacteria to a multifactorial condition involving complex interactions between host and microbial factors [1]. It is a prevalent disease that affects approximately 31.3% of Koreans [2]. Risk factors for CP include genetic predisposition and environmental conditions, such as smoking, which exacerbate inflammation [3]. The systemic consequences of CP are increasingly recognized, with evidence linking it to various chronic diseases such as cardiovascular and metabolic disorders [4,5,6,7].

Benign prostatic hyperplasia (BPH), characterized by prostate enlargement and lower urinary tract symptoms, affects up to 70–80% of aging males [8,9]. Inflammation and metabolic disturbances are known contributors to BPH, with inflammatory responses and cytokines playing key roles in hyperplastic changes in the prostate [10,11]. Since CP and BPH share an inflammatory pathophysiology, it has been proposed that CP may influence BPH risk. Several studies have reported an association between CP and increased BPH risk [12,13]. However, other studies have shown no clear relationship. For instance, a Mendelian randomization analysis found no difference in BPH rates between severe and non-severe periodontitis groups [14], and a meta-analysis reported no significant association between periodontal disease and prostate inflammation [15]. These conflicting results may originate from differences in the study population and multiple contributing factors for BPH, which may act as confounders. To overcome these issues, representative cohort data and adjustment or subgroup analyses for multiple covariables may be needed.

With the background of the prevalence of CP in Korea is relatively high and the aging population in Korea is increasing, with a corresponding rise in BPH prevalence in Korea, this study aims to address gaps in previous research by utilizing a nationwide, population-based cohort with a precisely matched nested case–control design, offering a more comprehensive analysis of the relationship between CP and BPH. Korea’s National Health Insurance Service (NHIS) provides a comprehensive, population-based database with high-quality health screening data, allowing for a robust and detailed analysis of this relationship. Unlike earlier studies, this research incorporates adjustments for confounding factors and explores subgroup analyses based on demographic, socioeconomic, and comorbidity variables to assess how CP frequency affects BPH risk. By addressing limitations in previous meta-analyses and population-specific findings, this study provides novel insights into the potential contribution of CP to BPH and the influence of patient-specific factors on this relationship.

## 2. Materials and Methods

### 2.1. Ethics

The present study was approved by the Ethics Committee of Hallym University (approval number: 2022-12-005). This study utilized data from the Korean National Health Insurance Service-Health Screening Cohort (2002–2019), which is a secondary dataset collected and managed by the Korean National Health Insurance Service. As the data were anonymized and de-identified before being provided to the researchers, obtaining written informed consent from participants was not feasible. Consequently, the requirement for informed consent was waived by the Ethics Committee.

### 2.2. Exposure (Chronic Periodontitis)

Participants diagnosed with CP were identified using the International Classification of Diseases, 10th Revision (ICD-10) code K05.3 [16]. These diagnoses were based on records of clinical visits where CP was documented [16]. The use of ICD-10 codes is a standard practice in large-scale population-based studies, as it ensures consistency and facilitates comprehensive data collection. While the ICD-10 codes alone were used to identify CP, potential misclassification bias may arise due to variability in clinical coding practices or subclinical cases that may go undiagnosed. To mitigate this, the study included participants with multiple clinical visits associated with CP diagnoses, increasing the likelihood that cases reflected true chronic conditions. Furthermore, ICD-10 codes are assigned by licensed healthcare professionals as part of routine clinical practice, which supports their reliability for population-level studies.

### 2.3. Outcome (Benign Prostate Hyperplasia)

Participants diagnosed with BPH were identified using the ICD-10 code N40, along with claim codes for procedures or examinations related to BPH (C4280, E7050, EY521, EY522, and EB451) [17]. Only participants who had visited a clinic at least twice with a BPH diagnosis were included to enhance diagnostic specificity and reduce potential misclassification bias.

Despite these measures, potential misclassification bias in BPH diagnosis cannot be entirely excluded. Cases of mild or asymptomatic BPH may not be captured, and coding practices may vary among healthcare providers. However, the requirement for multiple clinical visits and the use of specific claim codes associated with diagnostic or therapeutic procedures improve diagnostic accuracy and reduce the likelihood of false positives in identifying BPH cases.

### 2.4. Participant Selection

Participants with BPH were drawn from the Korean National Health Insurance Service-Health Screening Cohort (NHIS-HEALS), covering the years 2002 to 2019 (*n* = 97,314). The control group consisted of participants without a BPH diagnosis during the same period (*n* = 417,552).

To ensure accurate case definitions, BPH participants with a washout period of less than 2 years were excluded (*n* = 12,160). Additionally, control participants who had a single BPH diagnosis (*n* = 44,235) were excluded to minimize the risk of including individuals with potential misdiagnoses or transient symptoms that were not representative of true BPH. Participants with missing records of body mass index (BMI), fasting blood glucose, blood pressure, or total cholesterol were also removed (*n* = 10).

**Figure 1:**
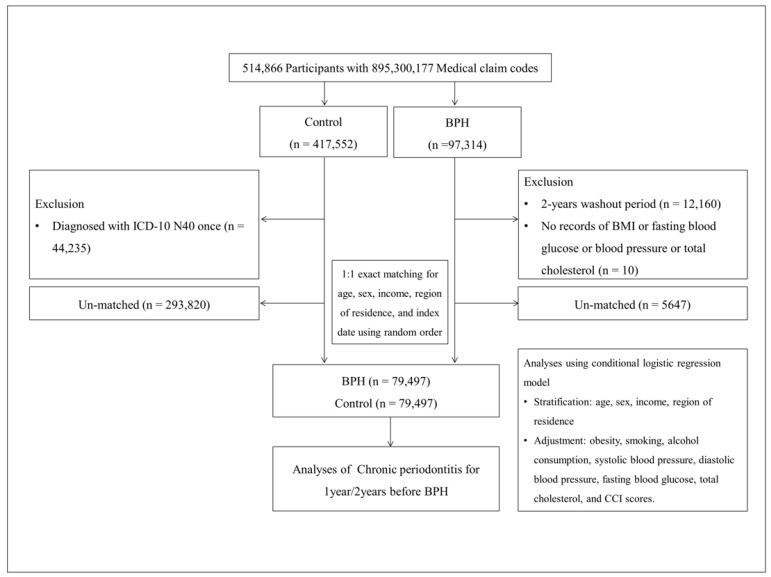
A schematic illustration of the participant selection process used in the present study. The flowchart outlines the inclusion and exclusion criteria at each step. BPH participants with a washout period of less than 2 years were excluded to ensure diagnostic accuracy, and control participants with a single BPH diagnosis were excluded to avoid potential misclassification. Participants with missing health data, including BMI, fasting blood glucose, blood pressure, or total cholesterol, were also removed. During the matching process, participants were excluded if they could not be matched due to demographic or clinical differences. Ultimately, of a total of 514,866 participants, 79,497 participants with BPH were matched with 79,497 control participants based on age, sex, income, and region of residence.

BPH participants and control participants were matched in a 1:1 ratio. During the matching procedure, 5647 BPH participants and 293,820 control participants were excluded due to unmatched demographic or clinical characteristics. Ultimately, 79,497 BPH participants were successfully matched with 79,497 control participants (Figure 1).

### 2.5. Covariates

Participants were categorized into 10 age groups and 5 income groups, with residential areas classified as urban or rural. Smoking habits, alcohol use, and obesity (based on BMI in kg/m^2^) were included as covariates due to their established associations with chronic inflammation and systemic health outcomes, which could potentially confound the relationship between CP and BPH.

Health screening parameters included systolic and diastolic blood pressure (mmHg), fasting blood glucose (mg/dL), total cholesterol (mg/dL), and hemoglobin (g/dL), as these are critical markers of metabolic and cardiovascular health that may influence BPH risk. Additionally, comorbidities were assessed using the Charlson Comorbidity Index (CCI) to account for the overall burden of chronic disease, which could confound the observed associations. These covariates were selected based on their relevance to inflammation, metabolic dysfunction, and other pathways that may link CP to BPH.

### 2.6. Statistical Analyses

Conditional logistic regression was performed to calculate odds ratios (ORs) and their 95% confidence intervals (CIs) for the association between CP and BPH. Participants with CP were categorized based on the frequency of CP diagnoses: CP ≥ 1, CP ≥ 2, and CP ≥ 3 within 1 year, and CP ≥ 1 within 2 years. These thresholds were chosen to capture varying levels of exposure to CP and their potential impact on BPH risk, reflecting the hypothesis that a higher frequency of CP may indicate greater systemic inflammation. Propensity score overlap-weighted multivariable logistic regressions were employed to estimate the crude (unadjusted) and overlap-weighted (adjusted for all covariates) odds ratios (ORs) and 95% confidence intervals (CIs) for incident BPH, accounting for the frequency of CP while adjusting for potential confounders.

Secondary analyses were performed by stratifying participants based on adjusted factors, including age, income, residential area, smoking status, alcohol use, obesity, blood pressure, fasting blood glucose, total cholesterol, and CCI. These stratifications allowed us to evaluate whether the association between CP and BPH differed across subgroups with varying demographic, socioeconomic, lifestyle, and health characteristics.

To assess the robustness of the findings, sensitivity analyses were conducted by varying key inclusion criteria, such as excluding participants with a single CP diagnosis to minimize potential misclassification, and adjusting for additional variables not included in the primary analysis. These analyses confirmed the consistency of the observed associations. A *p*-value of <0.05 was considered statistically significant. All statistical analyses were conducted using SAS software, version 9.4 (SAS Institute Inc., Cary, NC, USA).

## 3. Results

The average number of CPs within 1 year was 0.66 for the BPH group and 1.52 for the control group (Table 1). The average number of CPs within 2 years was 1.24 for the BPH group and 0.98 for the control group. BMI categories, patterns of smoking and alcohol intake, blood pressure measurements (systolic and diastolic), fasting glucose levels, total cholesterol concentrations, and CCI values showed differences when comparing individuals with BPH to those in the control group.

Participants with CP ≥ 1 within 1 year were significantly more likely to have BPH, with an OR of 1.34 (95% CI = 1.31–1.37, *p* < 0.001; Table 2). This means that individuals with at least one CP diagnosis within a year had a 34% higher likelihood of being diagnosed with BPH compared to those without CP during the same period. This association was consistent across all subgroups, including age, income, residential region, and health-related variables. Among these subgroups, the low-income group showed the highest odds of BPH (OR = 1.43, 95% CI = 1.38–1.48, *p* < 0.001), indicating a 43% higher likelihood of BPH among low-income individuals with CP compared to those without CP.

**Figure 2:**
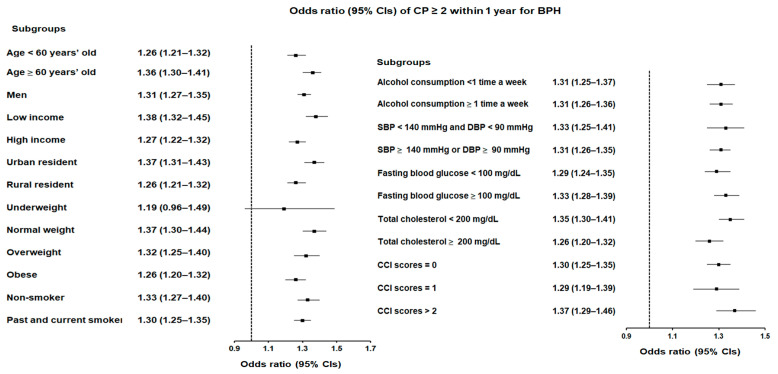
Forest plots illustrating the adjusted odds ratio and corresponding 95% confidence intervals (CIs) for demographic, lifestyle, and comorbid factors in relation to chronic periodontitis (CP) for incident benign prostatic hyperplasia (BPH) when participants are diagnosed with CP ≥ 2 within 1 year before the index date.

**Figure 3:**
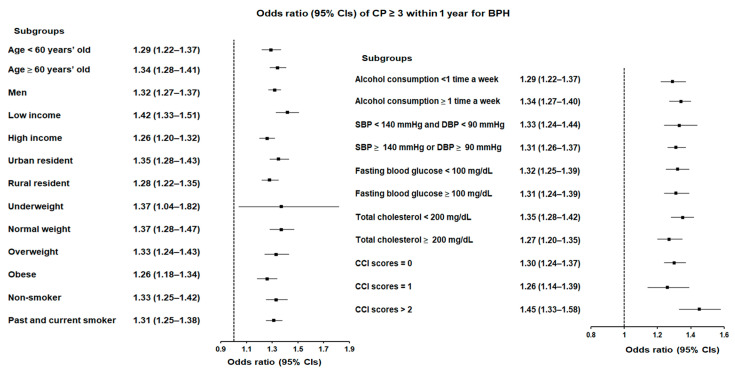
Forest plots illustrating the adjusted odds ratio and corresponding 95% confidence intervals (CIs) for demographic, lifestyle, and comorbid factors in relation to chronic periodontitis (CP) for incident benign prostatic hyperplasia (BPH) when participants are diagnosed with CP ≥ 3 within 1 year before the index date.

A similar pattern was observed for participants with CP ≥ 2 and CP ≥ 3 within 1 year. For participants with CP ≥ 2, the likelihood of BPH increased by 31% (OR = 1.31, 95% CI = 1.28–1.35, *p* < 0.001; Figure 2 and Supplementary Table S1), with the low-income group again showing the greatest risk (OR = 1.38, 95% CI = 1.32–1.45, *p* < 0.001). Participants with CP ≥ 3 within 1 year showed a slightly higher likelihood of BPH, with a 32% increased risk (OR = 1.32, 95% CI = 1.27–1.37, *p* < 0.001; Figure 3 and Supplementary Table S2). The highest risk in this group was observed in individuals with a CCI score ≥ 2 (OR = 1.45, 95% CI = 1.33–1.58, *p* < 0.001), meaning that patients with multiple comorbidities and frequent CP episodes had a 45% greater likelihood of BPH compared to those without CP.

**Figure 4:**
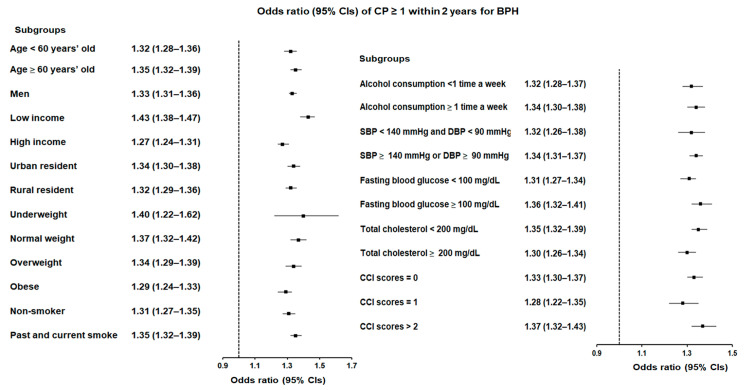
Forest plots illustrating the adjusted odds ratio and corresponding 95% confidence intervals (CIs) for demographic, lifestyle, and comorbid factors in relation to chronic periodontitis (CP) for incident benign prostatic hyperplasia (BPH) when participants are diagnosed with CP ≥ 1 within 2 years before the index date.

The association between CP frequency and BPH was also observed over a 2-year period. Participants with CP ≥ 1 within 2 years had a 34% increased likelihood of BPH (OR = 1.34, 95% CI = 1.31–1.37, *p* < 0.001; Figure 4 and Supplementary Table S3). The low-income group in this category again demonstrated the highest risk, with a 43% higher likelihood of BPH (OR = 1.43, 95% CI = 1.38–1.47, *p* < 0.001).

## 4. Discussion

A history of recurrent CP episodes was associated with an increased occurrence of BPH in the adult male population in Korea. There was no dose-dependent association between the frequency of CP history and the occurrence of BPH in our study. However, the low-income group demonstrated the greatest association between CP history and the occurrence of BPH. Additionally, among participants with the most frequent CP history (≥3 within 1 year), those with a high number of comorbidities (CCI score ≥ 2) showed the strongest correlation between CP and BPH. These findings highlight the importance of addressing CP as a potential risk factor for BPH, especially in vulnerable populations, such as those with low socioeconomic status or high comorbidities. From a clinical perspective, practitioners need to consider incorporating routine periodontal health assessments into the care of patients at risk for BPH. Early detection and management of CP may help reduce systemic inflammation, potentially mitigating its contribution to BPH development.

The association between CP and prostate disease has been suggested in previous studies [12,13,18]. For example, a health claim database study in Taiwan reported a 2.59- to 2.64-fold greater risk of prostate disorders in patients with CP [13]. Similarly, a cross-sectional study demonstrated 4.83-fold greater odds of BPH in patients with periodontitis [12]. These findings suggest a strong link between CP and prostate disease; however, our study identified comparatively lower odds of BPH associated with CP. This discrepancy may be attributed to methodological differences, such as the use of a strictly matched control group and the comprehensive adjustment of numerous covariables in our study, which reduced potential confounding effects. Additionally, unlike previous studies, our research varied the frequency of CP and analyzed its association with BPH but found no evidence of a dose-dependent relationship. This suggests that factors other than CP frequency alone may influence the risk of BPH.

Conflicting evidence from Mendelian randomization studies and meta-analyses has questioned the association between CP and BPH [14,15]. For example, an epidemiological study of 40 men who underwent health check-ups found no statistically significant differences in the prevalence of clinical BPH between severe and non-severe periodontitis groups, nor in the prevalence of severe periodontitis between individuals with and without BPH [14]. Similarly, Mendelian randomization analysis of genetic data from the FinnGen project, which included BPH (13,118 cases and 72,799 controls) and periodontitis (3046 cases and 195,395 controls), provided no evidence of a causal link between periodontitis and BPH [14]. A meta-analysis of four observational cohort studies and three case–control studies also concluded that periodontal disease does not increase the risk of prostate inflammation [15]. These inconsistencies may arise from differences in study populations, methodologies, and the inability to fully control for confounders, such as lifestyle factors or underlying health conditions. Our study sought to address these limitations by employing a nationwide cohort with a rigorously matched case–control design and adjusting for key confounding variables.

Potential mechanisms linking CP to BPH may involve systemic inflammation, microbiota changes, and immune responses. Chronic periodontitis is known to contribute to systemic inflammation through the release of inflammatory mediators such as interleukin-6 (IL-6) and tumor necrosis factor-alpha (TNF-α) [19], which are also implicated in prostate hyperplasia [20]. Animal studies have demonstrated changes in gut microbiota composition in models combining periodontitis and BPH, suggesting that microbiota-mediated metabolic alterations could link these conditions [21]. Fusobacterium nucleatum, a periodontal pathogen, has been detected in whole-mount prostatectomy samples from patients with BPH, further supporting the hypothesis of microbial dissemination contributing to prostate inflammation [22].

Additionally, a review has highlighted the potential role of oral pathogens and periodontal inflammatory mediators in prostate cancer development, which may share pathophysiological mechanisms with BPH [23]. These findings suggest that systemic inflammation, driven by oral pathogens and altered microbiota, could mediate the observed association between CP and BPH. However, the absence of a dose-dependent relationship in our study indicates that these mechanisms may be modulated by individual susceptibility or other factors, such as genetic predisposition, immune system variability, or environmental influences.

In the present study, CP patients with low income or a greater number of comorbidities had significantly higher odds of BPH compared to those in other subgroups. This elevated risk in low-income individuals may be attributed to disparities in healthcare access, preventative services, and oral healthcare, which can result in delayed diagnosis and treatment of CP, exacerbating systemic inflammation. Socioeconomic status has been shown in previous studies to influence the risk of CP, as low-income status is associated with increased susceptibility to CP and poor health outcomes [24,25].

A greater number of comorbidities may further increase the risk of developing BPH, as comorbid conditions often contribute to systemic inflammation and other chronic disease processes. Additionally, low-income populations may face greater exposure to risk factors such as smoking, poor diet, and limited resources for managing chronic conditions, all of which contribute to systemic inflammation. Chronic stress and occupational factors prevalent in disadvantaged populations may also amplify inflammatory pathways, further linking CP to BPH risk. Given these findings, CP patients with low income or comorbidities require meticulous evaluation and management to prevent or detect the possible occurrence of BPH early.

This study analyzed data from a large nationwide cohort. The large sample size and unbiased selection of control participants strengthened the reliability of the statistical analysis. Additionally, the analysis was adjusted for various potential confounding factors, such as demographic characteristics, socioeconomic status, lifestyle behaviors, and comorbidities.

However, this study has several limitations that warrant discussion. First, the reliance on ICD-10 codes for identifying CP and BPH diagnoses may have introduced potential misclassification bias. Although ICD-10 codes are widely used and assigned by trained healthcare professionals, they lack clinical validation for disease severity, type, or treatment details. As a result, subclinical or undiagnosed cases may not have been captured, potentially leading to an underestimation of the true prevalence of CP or BPH. Second, the study did not include direct measures of inflammation or biomarkers that could further elucidate the pathophysiological link between CP and BPH. Third, while the analysis adjusted for numerous confounders, other potential factors related to BPH, such as dietary habits, a history of prostatitis, or genetic predisposition, were not included due to the limitations of health claim data. These unmeasured variables could influence the observed associations. Fourth, the exclusion of participants with incomplete health data and the use of a matched cohort design, while enhancing internal validity, may introduce selection bias. By excluding participants with missing data, the study sample may not fully represent the general population, particularly individuals from underserved groups who may have incomplete health records. Furthermore, the matching process may limit the variability in the cohort, potentially reducing the generalizability of findings to broader populations or settings. Finally, the retrospective study design inherently limits the ability to establish a causal relationship between CP and BPH. The findings demonstrate an association but cannot confirm whether CP directly contributes to the development of BPH or whether shared underlying factors drive both conditions. Future studies should consider incorporating clinical validation of diagnoses and directly measuring disease severity through biomarkers or patient-reported outcomes to reduce the risk of misclassification and variability. Despite these limitations, this study provides valuable evidence supporting the association of CP with BPH, particularly among individuals with low economic status and high comorbidities.

## 5. Conclusions

A history of CP within the past year was associated with an increased incidence of BPH among adult male Koreans. This association was particularly evident in individuals with low socioeconomic status and those with comorbid conditions, highlighting the importance of addressing these risk factors in clinical practice. These findings suggest that incorporating periodontal health assessments into routine care for individuals at risk for BPH may help mitigate systemic inflammation and potentially reduce BPH risk. Future studies need to focus on elucidating the mechanisms linking CP and BPH, such as the role of systemic inflammation, microbiota changes, or genetic predisposition, to better understand their pathophysiological relationship.

## Figures and Tables

**Table 1:** The variation in demographic and clinical features.

Characteristics		Total Participants		
		BPH	Control	Standardized Difference
Age (years old) (n, %)				0.00
	40–44	557 (0.70)	557 (0.70)	
	45–49	4989 (6.28)	4989 (6.28)	
	50–54	13,040 (16.40)	13,040 (16.40)	
	55–59	17,841 (22.44)	17,841 (22.44)	
	60–64	16,847 (21.19)	16,847 (21.19)	
	65–69	14,294 (17.98)	14,294 (17.98)	
	70–74	7681 (9.66)	7681 (9.66)	
	75–79	3014 (3.79)	3014 (3.79)	
	80–84	991 (1.25)	991 (1.25)	
	85+	243 (0.31)	243 (0.31)	
Sex (n, %)				0.00
	Male	79,497 (100.0)	79,497 (100.0)	
Income (n, %)				0.00
	1 (lowest)	10,797 (13.58)	10,797 (13.58)	
	2	9737 (12.25)	9737 (12.25)	
	3	12,162 (15.30)	12,162 (15.30)	
	4	17,356 (21.83)	17,356 (21.83)	
	5 (highest)	29,445 (37.04)	29,445 (37.04)	
Region of residence (n, %)				0.00
	Urban	36,288 (45.65)	36,288 (45.65)	
	Rural	43,209 (54.35)	43,209 (54.35)	
Obesity ^†^ (n, %)				0.11
	Underweight	1511 (1.90)	2279 (2.87)	
	Normal	24,946 (31.38)	27,847 (35.03)	
	Overweight	23,651 (29.75)	22,262 (28.00)	
	Obese I	27,604 (34.72)	25,381 (31.93)	
	Obese II	1785 (2.25)	1728 (2.17)	
Smoking status (n, %)				0.22
	Nonsmoker	36,299 (45.66)	32,508 (40.89)	
	Past smoker	22,443 (28.23)	18,551 (23.34)	
	Current smoker	20,755 (26.11)	28,438 (35.77)	
Alcohol consumption (n, %)				0.09
	<1 time a week	39,014 (49.08)	35,653 (44.85)	
	≥1 time a week	40,483 (50.92)	43,844 (55.15)	
Systolic blood pressure (n, %)				0.09
	<120 mmHg	21,234 (26.71)	19,840 (24.96)	
	120–139 mmHg	41,556 (52.27)	40,120 (50.47)	
	≥140 mmHg	16,707 (21.02)	19,537 (24.58)	
Diastolic blood pressure (n, %)				0.07
	<80 mmHg	33,562 (42.22)	31,614 (39.77)	
	80–89 mmHg	31,230 (39.28)	31,023 (39.02)	
	≥90 mmHg	14,705 (18.50)	16,860 (21.21)	
Fasting blood glucose (n, %)				0.04
	<100 mg/dL	45,407 (57.12)	44,211 (55.61)	
	100–125 mg/dL	25,202 (31.70)	25,455 (32.02)	
	≥126 mg/dL	8888 (11.18)	9831 (12.37)	
Total cholesterol (n, %)				0.02
	<200 mg/dL	46,000 (57.86)	45,601 (57.36)	
	200–239 mg/dL	24,829 (31.23)	25,080 (31.55)	
	≥240 mg/dL	8668 (10.90)	8816 (11.09)	
CCI score (n, %)				0.05
	0	44,090 (55.46)	48,571 (61.10)	
	1	13,656 (17.18)	11,575 (14.56)	
	≥2	21,751 (27.36)	19,351 (24.34)	
The number of CP (Mean, Sd)				
	within 1 year	0.66 (1.54)	0.51 (1.33)	0.10
	within 2 years	1.24 (2.40)	0.98 (2.06)	0.12

Abbreviations: BPH, Benign prostatic hyperplasia; CCI, Charlson comorbidity index; CP, Chronic periodontitis; Sd, Standard deviation. ^†^ Obesity (BMI, body mass index, kg/m^2^) was categorized as <18.5 (underweight), ≥18.5 to <23 (normal), ≥23 to <25 (overweight), ≥25 to <30 (obese I), and ≥30 (obese II).

**Table 2:** Crude and adjusted odd ratios of CP for BPH when participants are diagnosed with CP ≥ 1 within 1 year before index date.

Characteristics		N of BPH	N of Control	Odd Ratios for BPH (95% Confidence Interval)					
		(Exposure/Total, %)	(Exposure/Total, %)	Crude ^†^	*p*-Value	Model 1 ^†,‡^	*p*-Value	Model 2 ^†,§^	*p*-Value
Total (n = 158,994)									
	CP < 1	57,362/79,497 (72.2%)	61,710/79,497 (77.6%)	1		1		1	
	CP ≥ 1	22,135/79,497 (27.8%)	17,787/79,497 (22.4%)	1.34 (1.31–1.37)	<0.001 *	1.35 (1.32–1.38)	<0.001 *	1.34 (1.31–1.37)	<0.001 *
Age < 60 years old (n = 72,854)									
	CP < 1	26,691/36,427 (73.3%)	28,440/36,427 (78.1%)	1		1		1	
	CP ≥ 1	9736/36,427 (26.7%)	7987/36,427 (21.9%)	1.30 (1.26–1.34)	<0.001 *	1.30 (1.25–1.34)	<0.001 *	1.31 (1.27–1.36)	<0.001 *
Age ≥ 60 years old (n = 86,140)									
	CP < 1	30,671/43,070 (71.2%)	33,270/43,070 (77.3%)	1		1		1	
	CP ≥ 1	12,399/43,070 (28.8%)	9800/43,070 (22.8%)	1.37 (1.33–1.42)	<0.001 *	1.37 (1.33–1.41)	<0.001 *	1.36 (1.31–1.40)	<0.001 *
Men (n = 158,994)									
	CP < 1	57,362/79,497 (72.2%)	61,710/79,497 (77.6%)	1		1		1	
	CP ≥ 1	22,135/79,497 (27.8%)	17,787/79,497 (22.4%)	1.34 (1.31–1.37)	<0.001 *	1.33 (1.30–1.36)	<0.001 *	1.33 (1.30–1.36)	<0.001 *
Low income (n = 65,392)									
	CP < 1	23,793/32,696 (72.8%)	25,985/32,696 (79.5%)	1		1		1	
	CP ≥ 1	8903/32,696 (27.2%)	6711/32,696 (20.5%)	1.45 (1.40–1.50)	<0.001 *	1.44 (1.39–1.49)	<0.001 *	1.43 (1.38–1.48)	<0.001 *
High income (n = 93,602)									
	CP < 1	33,569/46,801 (71.7%)	35,725/46,801 (76.3%)	1		1		1	
	CP ≥ 1	13,232/46,801 (28.3%)	11,076/46,801 (23.7%)	1.27 (1.23–1.31)	<0.001 *	1.27 (1.23–1.30)	<0.001 *	1.27 (1.23–1.31)	<0.001 *
Urban residents (n = 72,576)									
	CP < 1	25,624/36,288 (70.6%)	27,836/36,288 (76.7%)	1		1		1	
	CP ≥ 1	10,664/36,288 (29.4%)	8452/36,288 (23.3%)	1.37 (1.33–1.42)	<0.001 *	1.36 (1.31–1.41)	<0.001 *	1.36 (1.32–1.41)	<0.001 *
Rural residents (n = 86,418)									
	CP < 1	31,738/43,209 (73.5%)	33,874/43,209 (78.4%)	1		1		1	
	CP ≥ 1	11,471/43,209 (26.6%)	9335/43,209 (21.6%)	1.31 (1.27–1.35)	<0.001 *	1.31 (1.27–1.35)	<0.001 *	1.30 (1.26–1.35)	<0.001 *
Underweight (n = 3790)									
	CP < 1	1166/1511 (77.2%)	1867/2279 (81.9%)	1		1		1	
	CP ≥ 1	345/1511 (22.8%)	412/2279 (18.1%)	1.34 (1.14–1.57)	<0.001 *	1.33 (1.13–1.56)	0.001 *	1.32 (1.12–1.55)	0.001 *
Normal weight (n = 52,793)									
	CP < 1	18,325/24,946 (73.5%)	22,100/27,847 (79.4%)	1		1		1	
	CP ≥ 1	6621/24,946 (26.5%)	5747/27,847 (20.6%)	1.39 (1.33–1.45)	<0.001 *	1.38 (1.32–1.44)	<0.001 *	1.39 (1.33–1.44)	0.001 *
Overweight (n = 45,913)									
	CP < 1	16,951/23,651 (71.7%)	17,105/22,262 (76.8%)	1		1		1	
	CP ≥ 1	6700/23,651 (28.3%)	5157/22,262 (23.2%)	1.31 (1.26–1.37)	<0.001 *	1.30 (1.24–1.35)	<0.001 *	1.31 (1.26–1.37)	<0.001 *
Obese (n = 56,498)									
	CP < 1	20,920/29,389 (71.2%)	20,638/27,109 (76.1%)	1		1		1	
	CP ≥ 1	8469/29,389 (28.8%)	6471/27,109 (23.9%)	1.29 (1.24–1.34)	<0.001 *	1.28 (1.23–1.33)	<0.001 *	1.30 (1.25–1.35)	<0.001 *
Non-smoker (n = 68,807)									
	CP < 1	27,156/36,299 (74.8%)	25,874/32,508 (79.6%)	1		1		1	
	CP ≥ 1	9143/36,299 (25.2%)	6634/32,508 (20.4%)	1.31 (1.27–1.36)	<0.001 *	1.31 (1.27–1.36)	<0.001 *	1.32 (1.27–1.37)	<0.001 *
Past and current smoker (n = 90,187)									
	CP < 1	30,206/43,198 (69.9%)	35,836/46,989 (76.3%)	1		1		1	
	CP ≥ 1	12,992/43,198 (30.1%)	11,153/46,989 (23.7%)	1.38 (1.34–1.42)	<0.001 *	1.37 (1.33–1.41)	<0.001 *	1.34 (1.30–1.38)	<0.001 *
Alcohol consumption < 1 time a week (n = 74,667)									
	CP < 1	29,057/39,014 (74.5%)	28,398/35,653 (79.7%)	1		1		1	
	CP ≥ 1	9957/39,014 (25.5%)	7255/35,653 (20.4%)	1.34 (1.30–1.39)	<0.001 *	1.34 (1.29–1.39)	<0.001 *	1.33 (1.29–1.38)	<0.001 *
Alcohol consumption ≥ 1 time a week (n = 84,327)									
	CP < 1	28,305/40,483 (69.9%)	33,312/43,844 (76.0%)	1		1		1	
	CP ≥ 1	12,178/40,483 (30.1%)	10,532/43,844 (24.0%)	1.36 (1.32–1.40)	<0.001 *	1.35 (1.31–1.39)	<0.001 *	1.33 (1.29–1.38)	<0.001 *
SBP < 120 mmHg and DBP < 80 mmHg (n = 36,504)									
	CP < 1	13,196/18,789 (70.2%)	13,457/17,715 (76.0%)	1		1		1	
	CP ≥ 1	5593/18,789 (29.8%)	4258/17,715 (24.0%)	1.34 (1.28–1.40)	<0.001 *	1.34 (1.28–1.41)	<0.001 *	1.34 (1.28–1.41)	<0.001 *
SBP ≥ 120 mmHg or DBP ≥ 80 mmHg (n = 122,490)									
	CP < 1	44,166/60,708 (72.8%)	48,253/61,782 (78.1%)	1		1		1	
	CP ≥ 1	16,542/60,708 (27.3%)	13,529/61,782 (21.9%)	1.34 (1.30–1.37)	<0.001 *	1.33 (1.29–1.36)	<0.001 *	1.33 (1.29–1.36)	<0.001 *
Fasting blood glucose < 100 mg/dL (n = 89,618)									
	CP < 1	33,366/45,407 (73.5%)	34,631/44,211 (78.3%)	1		1		1	
	CP ≥ 1	12,041/45,407 (26.5%)	9580/44,211 (21.7%)	1.30 (1.27–1.35)	<0.001 *	1.30 (1.26–1.34)	<0.001 *	1.30 (1.26–1.34)	<0.001 *
Fasting blood glucose ≥ 100 mg/dL (n = 69,376)									
	CP < 1	23,996/34,090 (70.4%)	27,079/35,286 (76.7%)	1		1		1	
	CP ≥ 1	10,094/34,090 (29.6%)	8207/35,286 (23.3%)	1.39 (1.34–1.44)	<0.001 *	1.37 (1.33–1.42)	<0.001 *	1.37 (1.33–1.42)	<0.001 *
Total cholesterol < 200 mg/dL (n = 91,601)									
	CP < 1	33,102/46,000 (72.0%)	35,428/45,601 (77.7%)	1		1		1	
	CP ≥ 1	12,898/46,000 (28.0%)	10,173/45,601 (22.3%)	1.36 (1.32–1.40)	<0.001 *	1.35 (1.31–1.39)	<0.001 *	1.34 (1.30–1.39)	<0.001 *
Total cholesterol ≥ 200 mg/dL (n = 67,393)									
	CP < 1	24,260/33,497 (72.4%)	26,282/33,896 (77.5%)	1		1		1	
	CP ≥ 1	9237/33,497 (27.6%)	7614/33,896 (22.5%)	1.31 (1.27–1.36)	<0.001 *	1.31 (1.26–1.35)	<0.001 *	1.31 (1.27–1.36)	<0.001 *
CCI scores = 0 (n = 92,661)									
	CP < 1	31,254/44,090 (70.9%)	37,220/48,571 (76.6%)	1		1		1	
	CP ≥ 1	12,836/44,090 (29.1%)	11,351/48,571 (23.4%)	1.35 (1.31–1.39)	<0.001 *	1.34 (1.31–1.39)	<0.001 *	1.34 (1.30–1.38)	<0.001 *
CCI score = 1 (n = 25,231)									
	CP < 1	9925/13,656 (72.7%)	8958/11,575 (77.4%)	1		1		1	
	CP ≥ 1	3731/13,656 (27.3%)	2617/11,575 (22.6%)	1.29 (1.21–1.36)	<0.001 *	1.28 (1.21–1.35)	<0.001 *	1.26 (1.19–1.34)	<0.001 *
CCI score ≥ 2 (n = 41,102)									
	CP < 1	16,183/21,751 (74.4%)	15,532/19,351 (80.3%)	1		1		1	
	CP ≥ 1	5568/21,751 (25.6%)	3819/19,351 (19.7%)	1.40 (1.34–1.47)	<0.001 *	1.39 (1.33–1.46)	<0.001 *	1.37 (1.31–1.44)	<0.001 *

Abbreviations: SBP, Systolic blood pressure; DBP, Diastolic blood pressure; CCI, Charlson Comorbidity Index; * Conditional or unconditional logistic regression analysis, Significance at *p* < 0.05; ^†^ Stratified model for age, sex, income, and region of residence. ^‡^ Model 1 was adjusted for smoking, alcohol consumption, obesity, and CCI scores. ^§^ Model 2 was adjusted for Model 1 plus total cholesterol, SBP, DBP, and fasting blood glucose.

## Data Availability

The data utilized in this study were obtained from the Korean National Health Insurance Service under a license agreement and are therefore not accessible to the public. However, the data can be provided by the authors upon reasonable request, subject to approval from the Korean National Health Insurance Service.

## Supplementary Materials

The following are available online at https://www.mdpi.com/article/10.3390/jcm14041279/s1, Table S1: Crude and adjusted odd ratios of CP for BPH when participants are diagnosed with CP ≥ 2 within 1 year before index date, Table S2: Crude and adjusted odd ratios of CP for BPH when participants are diagnosed with CP ≥ 3 within 1 year before index date, Table S3: Crude and adjusted odd ratios of CP for BPH when participants are diagnosed with CP ≥ 1 within 2 years before index date.

## References

[1] Abdulkareem A.A., Al-Taweel F.B., Al-Sharqi A.J.B., Gul S.S., Sha A., Chapple I.L.C. (2023). Current concepts in the pathogenesis of periodontitis: From symbiosis to dysbiosis. J. Oral Microbiol..

[2] Lee J.H., Lee J.S., Park J.Y., Choi J.K., Kim D.W., Kim Y.T., Choi S.H. (2015). Association of Lifestyle-Related Comorbidities with Periodontitis: A Nationwide Cohort Study in Korea. Medicine.

[3] Reynolds M.A. (2014). Modifiable risk factors in periodontitis: At the intersection of aging and disease. Periodontol. 2000.

[4] Cecoro G., Annunziata M., Iuorio M.T., Nastri L., Guida L. (2020). Periodontitis, Low-Grade Inflammation and Systemic Health: A Scoping Review. Medicina.

[5] Dietrich T., Garcia R.I. (2005). Associations between periodontal disease and systemic disease: Evaluating the strength of the evidence. J. Periodontol..

[6] Li P., He L., Sha Y.Q., Luan Q.X. (2009). Relationship of metabolic syndrome to chronic periodontitis. J. Periodontol..

[7] Naderi S., Merchant A.T. (2020). The Association Between Periodontitis and Cardiovascular Disease: An Update. Curr. Atheroscler. Rep..

[8] Chughtai B., Forde J.C., Thomas D.D., Laor L., Hossack T., Woo H.H., Te A.E., Kaplan S.A. (2016). Benign prostatic hyperplasia. Nat. Rev. Dis. Primers.

[9] Egan K.B. (2016). The Epidemiology of Benign Prostatic Hyperplasia Associated with Lower Urinary Tract Symptoms: Prevalence and Incident Rates. Urol. Clin. N. Am..

[10] Madersbacher S., Sampson N., Culig Z. (2019). Pathophysiology of Benign Prostatic Hyperplasia and Benign Prostatic Enlargement: A Mini-Review. Gerontology.

[11] Chughtai B., Lee R., Te A., Kaplan S. (2011). Inflammation and benign prostatic hyperplasia: Clinical implications. Curr. Urol. Rep..

[12] Wu L., Li B.H., Wang Y.Y., Wang C.Y., Zi H., Weng H., Huang Q., Zhu Y.J., Zeng X.T. (2019). Periodontal disease and risk of benign prostate hyperplasia: A cross-sectional study. Mil. Med. Res..

[13] Fu E., Cheng C.M., Chung C.H., Lee W.C., Chen W.L., Sun G.H., Chien W.C. (2021). Association of chronic periodontitis with prostatic hyperplasia and prostatitis: A population-based cohort study in Taiwan. J. Periodontol..

[14] Wei H., Tian G., Xu S., Du Y., Li M., Wang Y., Deng J., Quan C. (2024). Evaluation of bi-directional causal association between periodontitis and benign prostatic hyperplasia: Epidemiological studies and two-sample mendelian randomization analysis. Front. Genet..

[15] Ortiz de Urbina Comeron P., Zubizarreta-Macho A., Lobo Galindo A.B., Montiel-Company J.M., Lorenzo-Gomez M.F., Flores Fraile J. (2023). Relationship between Prostate Inflammation and Periodontal Disease-A Systematic Review and Meta-Analysis. J. Clin. Med..

[16] Byun S.H., Min C., Park I.S., Kim H., Kim S.K., Park B.J., Choi H.G., Hong S.J. (2020). Increased Risk of Chronic Periodontitis in Chronic Rhinosinusitis Patients: A Longitudinal Follow-Up Study Using a National Health-Screening Cohort. J. Clin. Med..

[17] Kim S.H., Kwon W.A., Joung J.Y. (2021). Impact of Benign Prostatic Hyperplasia and/or Prostatitis on the Risk of Prostate Cancer in Korean Patients. World J. Mens Health.

[18] Hyun H., Park Y.W., Kwon Y.C., Cho B.K., Lee J.H. (2021). Relationship Between Chronic Periodontitis and Lower Urinary Tract Symptoms/Benign Prostatic Hyperplasia. Int. Neurourol. J..

[19] Ramadan D.E., Hariyani N., Indrawati R., Ridwan R.D., Diyatri I. (2020). Cytokines and Chemokines in Periodontitis. Eur. J. Dent..

[20] Ogbonnaya U.C., Dappa B.D. (2023). Comparative Study of Interleukin- 6 (Il-6) and Tumour Necrosis Factor Alpha (Tnf-A) Levels in Prostate Cancer and Benign Prostatic Hyperplasia Subjects. J. Appl. Health Sci. Med..

[21] Guo X.P., Yang J., Wu L., Fang C., Gu J.M., Li F., Liu H.S., Li L.Y., Wang S.Y. (2023). Periodontitis relates to benign prostatic hyperplasia via the gut microbiota and fecal metabolome. Front. Microbiol..

[22] Alluri L.S.C., Paes Batista da Silva A., Verma S., Fu P., Shen D.L., MacLennan G., Gupta S., Bissada N.F. (2021). Presence of Specific Periodontal Pathogens in Prostate Gland Diagnosed with Chronic Inflammation and Adenocarcinoma. Cureus.

[23] da Silva A.P.B., Alluri L.S.C., Bissada N.F., Gupta S. (2019). Association between oral pathogens and prostate cancer: Building the relationship. Am. J. Clin. Exp. Urol..

[24] Schuch H.S., Peres K.G., Singh A., Peres M.A., Do L.G. (2017). Socioeconomic position during life and periodontitis in adulthood: A systematic review. Community Dent. Oral Epidemiol..

[25] Dickman S.L., Himmelstein D.U., Woolhandler S. (2017). Inequality and the health-care system in the USA. Lancet.

