# Supplementary material for: Chronic Periodontitis as a Risk Factor for Benign Prostatic Hyperplasia: A Cohort Study
Source: J Clin Med. 2025 Feb 14;14(4):1279. doi: 10.3390/jcm14041279 (PMC11857014; doi:10.3390/jcm14041279)
Supplement: Supplementary file 1 [file jcm-14-01279-s001.zip › jcm-3389538-supplementary.pdf]

Supplementary Table S1. Crude and adjusted odd ratios of CP for BPH when participants are diagnosed with CP ≥ 2 within 1 year before index date.

Characteristics	N of BPH	N of Control	Odd ratios for BPH (95% confidence interval)					
	(exposure/total, %)	(exposure/total, %)	Crude†	*P*-value	Model 1†‡	*P*-value	Model 2†§	*P*-value
Total (n = 158,994)								
CP < 2	67,988/79,497 (85.5%)	70,419/79,497 (88.6%)	1		1		1	
CP ≥ 2	11,509/79,497 (14.5%)	9,078/79,497 (11.4%)	1.32 (1.28–1.35)	<0.001*	1.32 (1.28–1.36)	<0.001*	1.31 (1.28–1.35)	<0.001*
Age < 60 years old (n = 72,854)								
CP < 2	31,441/36,427 (86.3%)	32,311/36,427 (88.7%)	1		1		1	
CP ≥ 2	4986/36,427 (13.7%)	4,116/36,427 (11.3%)	1.24 (1.19–1.30)	<0.001*	1.24 (1.19–1.30)	<0.001*	1.26 (1.21–1.32)	<0.001*
Age ≥ 60 years old (n = 86,140)								
CP < 2	36,547/43,070 (84.9%)	38,108/43,070 (88.5%)	1		1		1	
CP ≥ 2	6523/43,070 (15.2%)	4962/43,070 (11.5%)	1.37 (1.32–1.43)	<0.001*	1.36 (1.31–1.42)	<0.001*	1.36 (1.30–1.41)	<0.001*
Men (n = 158,994)								
CP < 2	67,988/79,497 (85.5%)	70,419/79,497 (88.6%)	1		1		1	
CP ≥ 2	11,509/79,497 (14.5%)	9078/79,497 (11.4%)	1.31 (1.27–1.35)	<0.001*	1.31 (1.27–1.35)	<0.001*	1.31 (1.27–1.35)	<0.001*
Low income (n = 65,392)								
CP < 2	28,123/32,696 (86.0%)	29,276/32,696 (89.5%)	1		1		1	
CP ≥ 2	4573/32,696 (14.0%)	3420/32,696 (10.5%)	1.39 (1.33–1.46)	<0.001*	1.38 (1.32–1.45)	<0.001*	1.38 (1.32–1.45)	<0.001*
High income (n = 93,602)								
CP < 2	39,865/46,801 (85.2%)	41,143/46,801 (87.9%)	1		1		1	
CP ≥ 2	6936/46,801 (14.8%)	5658/46,801 (12.1%)	1.27 (1.22–1.31)	<0.001*	1.26 (1.21–1.31)	<0.001*	1.27 (1.22–1.32)	<0.001*
Urban residents (n = 72,576)								
CP < 2	30,569/36,288 (84.2%)	31,925/36,288 (88.0%)	1		1		1	
CP ≥ 2	5719/36,288 (15.8%)	4363/36,288 (12.0%)	1.37 (1.31–1.43)	<0.001*	1.36 (1.30–1.42)	<0.001*	1.37 (1.31–1.43)	<0.001*
Rural residents (n = 86,418)								
CP < 2	37,419/43,209 (86.6%)	38,494/43,209 (89.1%)	1		1		1	
CP ≥ 2	5790/43,209 (13.4%)	4715/43,209 (10.9%)	1.26 (1.21–1.32)	<0.001*	1.26 (1.21–1.31)	<0.001*	1.26 (1.21–1.32)	<0.001*
Underweight (n = 3,790)								
CP < 2	1347/1511 (89.2%)	2070/2279 (90.8%)	1		1		1	
CP ≥ 2	164/1511 (10.9%)	209/2279 (9.2%)	1.21 (0.97–1.50)	0.089	1.20 (0.97–1.49)	0.098	1.19 (0.96–1.49)	0.113
Normal weight (n = 52,793)								
CP < 2	21,532/24,946 (86.3%)	24,949/27,847 (89.6%)	1		1		1	
CP ≥ 2	3414/24,946 (13.7%)	2898/27,847 (10.4%)	1.37 (1.30–1.44)	<0.001*	1.36 (1.29–1.43)	<0.001*	1.37 (1.30–1.44)	0.001*
Overweight (n = 45,913)								
CP < 2	20,109/23,651 (85.0%)	19,642/22,262 (88.2%)	1		1		1	
CP ≥ 2	3542/23,651 (15.0%)	2620/22,262 (11.8%)	1.32 (1.25–1.39)	<0.001*	1.31 (1.24–1.38)	<0.001*	1.32 (1.25–1.40)	<0.001*
Obese (n = 56,498)								
CP < 2	25,000/29,389 (85.1%)	23,758/27,109 (87.6%)	1		1		1	
CP ≥ 2	4389/29,389 (14.9%)	3351/27,109 (12.4%)	1.24 (1.19–1.31)	<0.001*	1.23 (1.17–1.29)	<0.001*	1.26 (1.20–1.32)	<0.001*
Non-smoker (n = 68,807)								
CP < 2	31,693/36,299 (87.3%)	29,283/32,508 (90.1%)	1		1		1	
CP ≥ 2	4606/36,299 (12.7%)	3225/32,508 (9.9%)	1.32 (1.26–1.38)	<0.001*	1.32 (1.26–1.39)	<0.001*	1.33 (1.27–1.40)	<0.001*
Past and current smoker (n = 90,187)								
CP < 2	36,295/43,198 (84.0%)	41,136/46,989 (87.5%)	1		1		1	
CP ≥ 2	6903/43,198 (16.0%)	5853/46,989 (12.5%)	1.34 (1.29–1.39)	<0.001*	1.33 (1.28–1.38)	<0.001*	1.30 (1.25–1.35)	<0.001*
Alcohol consumption < 1 time a week (n = 74,667)								
CP < 2	33,969/39,014 (87.1%)	32,037/35,653 (89.9%)	1		1		1	
CP ≥ 2	5045/39,014 (12.9%)	3616/35,653 (10.1%)	1.32 (1.26-1.38)	<0.001*	1.31 (1.25-1.37)	<0.001*	1.31 (1.25–1.37)	<0.001*
Alcohol consumption ≥ 1 time a week (n = 84,327)								

CP < 2	34,019/40,483 (84.0%)	38,382/43,844 (87.5%)	1		1		1	
CP ≥ 2	6464/40,483 (16.0%)	5462/43,844 (12.5%)	1.34 (1.28–1.39)	<0.001*	1.32 (1.27–1.38)	<0.001*	1.31 (1.26-1.36)	<0.001*
SBP < 120 mmHg and DBP < 80 mmHg (n = 36,504)								
CP < 2	15,868/18,789 (84.5%)	15,539/17,715 (87.7%)	1		1		1	
CP ≥ 2	2921/18,789 (15.6%)	2176/17,715 (12.3%)	1.31 (1.24–1.40)	<0.001*	1.32 (1.24–1.40)	<0.001*	1.33 (1.25–1.41)	<0.001*
SBP ≥ 120 mmHg or DBP ≥ 80 mmHg (n = 122,490)								
CP < 2	52,120/60,708 (85.9%)	54,880/61,782 (88.8%)	1		1		1	
CP ≥ 2	8588/60,708 (14.2%)	6902/61,782 (11.2%)	1.31 (1.27–1.36)	<0.001*	1.30 (1.26–1.35)	<0.001*	1.31 (1.26–1.35)	<0.001*
Fasting blood glucose < 100 mg/dL (n = 89,618)								
CP < 2	39,235/45,407 (86.4%)	39,412/44,211 (89.2%)	1		1		1	
CP ≥ 2	6172/45,407 (13.6%)	4799/44,211 (10.9%)	1.29 (1.24–1.34)	<0.001*	1.28 (1.23–1.33)	<0.001*	1.29 (1.24–1.35)	<0.001*
Fasting blood glucose ≥ 100 mg/dL (n = 69,376)								
CP < 2	28,753/34,090 (84.3%)	31,007/35,286 (87.9%)	1		1		1	
CP ≥ 2	5337/34,090 (15.7%)	4279/35,286 (12.1%)	1.35 (1.29–1.40)	<0.001*	1.33 (1.28–1.39)	<0.001*	1.33 (1.28–1.39)	<0.001*
Total cholesterol < 200mg/dL (n = 91,601)								
CP < 2	39,213/46,000 (85.3%)	40,453/45,601 (88.7%)	1		1		1	
CP ≥ 2	6787/46,000 (14.8%)	5148/45,601 (11.3%)	1.36 (1.31–1.41)	<0.001*	1.35 (1.30–1.41)	<0.001*	1.35 (1.30–1.41)	<0.001*
Total cholesterol ≥ 200mg/dL (n = 67,393)								
CP < 2	28,775/33,497 (85.9%)	29,966/33,896 (88.4%)	1		1		1	
CP ≥ 2	4722/33,497 (14.1%)	3930/33,896 (11.6%)	1.25 (1.20–1.31)	<0.001*	1.25 (1.19–1.30)	<0.001*	1.26 (1.20–1.32)	<0.001*
CCI scores = 0 (n = 92,661)								
CP < 2	37,382/44,090 (84.8%)	42,695/48,571 (87.9%)	1		1		1	
CP ≥ 2	6708/44,090 (15.2%)	5876/48,571 (12.1%)	1.30 (1.26–1.35)	<0.001*	1.30 (1.26–1.35)	<0.001*	1.30 (1.25–1.35)	<0.001*
CCI score = 1 (n = 25,231)								
CP < 2	11,672/13,656 (85.5%)	10,245/11,575 (88.5%)	1		1		1	
CP ≥ 2	1984/13,656 (14.5%)	1,330/11,575 (11.5%)	1.31 (1.22–1.41)	<0.001*	1.30 (1.21–1.40)	<0.001*	1.29 (1.19–1.39)	<0.001*
CCI score ≥ 2 (n = 41,102)								
CP < 2	18,934/21,751 (87.1%)	17,479/19,351 (90.3%)	1		1		1	
CP ≥ 2	2817/21,751 (13.0%)	1872/19,351 (9.7%)	1.39 (1.31–1.48)	<0.001*	1.38 (1.30–1.47)	<0.001*	1.37 (1.29–1.46)	<0.001*

Abbreviations: SBP, Systolic blood pressure; DBP, Diastolic blood pressure; CCI, Charlson Comorbidity Index; * Conditional or unconditional logistic regression analysis, Significance at $P < 0.05$. † Stratified model for age, sex, income, and region of residence. ‡ Model 1 was adjusted for smoking, alcohol consumption, obesity and CCI scores. § Model 2 was adjusted for model 1 plus total cholesterol, SBP, DBP, and fasting blood glucose.

Supplementary Table S2. Crude and adjusted odd ratios of CP for BPH when participants are diagnosed with CP ≥ 3 within 1 year before index date.

Characteristics	N of BPH	N of Control	Odd ratios for BPH (95% confidence interval)					
	(exposure/total, %)	(exposure/total, %)	Crude†	*P*-value	Model 1†‡	*P*-value	Model 2†§	*P*-value
Total (n = 158,994)								
CP < 3	72,797/79,497 (91.6%)	74,319/79,497 (93.5%)	1		1		1	
CP ≥ 3	6700/79,497 (8.4%)	5178/79,497 (6.5%)	1.32 (1.27–1.37)	<0.001*	1.33 (1.28–1.38)	<0.001*	1.32 (1.27–1.37)	<0.001*
Age < 60 years old (n = 72,854)								
CP < 3	31,441/36,427 (86.3%)	32,311/36,427 (88.7%)	1		1		1	
CP ≥ 3	4986/36,427 (13.7%)	4116/36,427 (11.3%)	1.27 (1.20–1.35)	<0.001*	1.27 (1.20–1.35)	<0.001*	1.29 (1.22–1.37)	<0.001*
Age ≥ 60 years old (n = 86,140)								
CP < 3	36,547/43,070 (84.9%)	38,108/43,070 (88.5%)	1		1		1	
CP ≥ 3	6523/43,070 (15.2%)	4962/43,070 (11.5%)	1.36 (1.29–1.43)	<0.001*	1.35 (1.29–1.42)	<0.001*	1.34 (1.28–1.41)	<0.001*
Men (n = 158,994)								
CP < 3	67,988/79,497 (85.5%)	70,419/79,497 (88.6%)	1		1		1	
CP ≥ 3	11,509/79,497 (14.5%)	9078/79,497 (11.4%)	1.32 (1.27–1.37)	<0.001*	1.31 (1.27–1.37)	<0.001*	1.32 (1.27–1.37)	<0.001*
Low income (n = 65,392)								
CP < 3	28,123/32,696 (86.0%)	29,276/32,696 (89.5%)	1		1		1	
CP ≥ 3	4573/32,696 (14.0%)	3420/32,696 (10.5%)	1.43 (1.34–1.52)	<0.001*	1.42 (1.33–1.51)	<0.001*	1.42 (1.33–1.51)	<0.001*
High income (n = 93,602)								
CP < 3	39,865/46,801 (85.2%)	41,143/46,801 (87.9%)	1		1		1	
CP ≥ 3	6936/46,801 (14.8%)	5658/46,801 (12.1%)	1.26 (1.20–1.32)	<0.001*	1.26 (1.20–1.32)	<0.001*	1.26 (1.20–1.32)	<0.001*
Urban residents (n = 72,576)								
CP < 3	30,569/36,288 (84.2%)	31,925/36,288 (88.0%)	1		1		1	
CP ≥ 3	5719/36,288 (15.8%)	4363/36,288 (12.0%)	1.36 (1.29–1.44)	<0.001*	1.35 (1.28–1.42)	<0.001*	1.35 (1.28–1.43)	<0.001*
Rural residents (n = 86,418)								
CP < 3	37,419/43,209 (86.6%)	38,494/43,209 (89.1%)	1		1		1	
CP ≥ 3	5790/43,209 (13.4%)	4715/43,209 (10.9%)	1.28 (1.22–1.35)	<0.001*	1.28 (1.21–1.35)	<0.001*	1.28 (1.22–1.35)	<0.001*
Underweight (n = 3790)								
CP < 3	1347/1,511 (89.2%)	2070/2279 (90.8%)	1		1		1	
CP ≥ 3	164/1511 (10.9%)	209/2,279 (9.2%)	1.37 (1.04–1.81)	0.024*	1.38 (1.04–1.81)	0.024*	1.37 (1.04–1.82)	0.026*
Normal weight (n = 52,793)								
CP < 3	21,532/24,946 (86.3%)	24,949/27,847 (89.6%)	1		1		1	
CP ≥ 3	3414/24,946 (13.7%)	2,898/27,847 (10.4%)	1.37 (1.28–1.47)	<0.001*	1.36 (1.27–1.46)	<0.001*	1.37 (1.28–1.47)	0.001*
Overweight (n = 45,913)								
CP < 3	20,109/23,651 (85.0%)	19,642/22,262 (88.2%)	1		1		1	
CP ≥ 3	3542/23,651 (15.0%)	2,620/22,262 (11.8%)	1.33 (1.24–1.43)	<0.001*	1.32 (1.23–1.41)	<0.001*	1.33 (1.24–1.43)	<0.001*
Obese (n = 56,498)								
CP < 3	25,000/29,389 (85.1%)	23,758/27,109 (87.6%)	1		1		1	
CP ≥ 3	4389/29,389 (14.9%)	3,351/27,109 (12.4%)	1.24 (1.17–1.32)	<0.001*	1.23 (1.16–1.31)	<0.001*	1.26 (1.18–1.34)	<0.001*
Non-smoker (n = 68,807)								
CP < 3	31,693/36,299 (87.3%)	29,283/32,508 (90.1%)	1		1		1	
CP ≥ 3	4606/36,299 (12.7%)	3,225/32,508 (9.9%)	1.31 (1.23–1.40)	<0.001*	1.32 (1.24–1.40)	<0.001*	1.33 (1.25–1.42)	<0.001*
Past and current smoker (n = 90,187)								
CP < 3	36,295/43,198 (84.0%)	41,136/46,989 (87.5%)	1		1		1	
CP ≥ 3	6903/43,198 (16.0%)	5,853/46,989 (12.5%)	1.36 (1.30–1.42)	<0.001*	1.35 (1.28–1.41)	<0.001*	1.31 (1.25–1.38)	<0.001*
Alcohol consumption < 1 time a week (n = 74,667)								
CP < 3	33,969/39,014 (87.1%)	32,037/35,653 (89.9%)	1		1		1	

CP ≥ 3	5045/39,014 (12.9%)	3,616/35,653 (10.1%)	1.30 (1.22–1.38)	<0.001*	1.29 (1.22–1.37)	<0.001*	1.29 (1.22–1.37)	<0.001*
Alcohol consumption ≥ 1 time a week (n = 84,327)								
CP < 3	34,019/40,483 (84.0%)	38,382/43,844 (87.5%)	1		1		1	
CP ≥ 3	6464/40,483 (16.0%)	5,462/43,844 (12.5%)	1.37 (1.30–1.43)	<0.001*	1.35 (1.29–1.42)	<0.001*	1.34 (1.27–1.40)	<0.001*
SBP < 120 mmHg and DBP < 80 mmHg (n = 36,504)								
CP < 3	15,868/18,789 (84.5%)	15,539/17,715 (87.7%)	1		1		1	
CP ≥ 3	2,921/18,789 (15.6%)	2,176/17,715 (12.3%)	1.32 (1.23–1.43)	<0.001*	1.32 (1.23–1.43)	<0.001*	1.33 (1.24–1.44)	<0.001*
SBP ≥ 120 mmHg or DBP ≥ 80 mmHg (n = 122,490)								
CP < 3	52,120/60,708 (85.9%)	54,880/61,782 (88.8%)	1		1		1	
CP ≥ 3	8,588/60,708 (14.2%)	6,902/61,782 (11.2%)	1.32 (1.26–1.38)	<0.001*	1.31 (1.25–1.37)	<0.001*	1.31 (1.26–1.37)	<0.001*
Fasting blood glucose < 100 mg/dL (n = 89,618)								
CP < 3	39,235/45,407 (86.4%)	39,412/44,211 (89.2%)	1		1		1	
CP ≥ 3	6,172/45,407 (13.6%)	4,799/44,211 (10.9%)	1.32 (1.25–1.39)	<0.001*	1.31 (1.24–1.38)	<0.001*	1.32 (1.25–1.39)	<0.001*
Fasting blood glucose ≥ 100 mg/dL (n = 69,376)								
CP < 3	28,753/34,090 (84.3%)	31,007/35,286 (87.9%)	1		1		1	
CP ≥ 3	5337/34,090 (15.7%)	4279/35,286 (12.1%)	1.33 (1.26–1.41)	<0.001*	1.32 (1.25–1.39)	<0.001*	1.31 (1.24–1.39)	<0.001*
Total cholesterol < 200mg/dL (n = 91,601)								
CP < 3	39,213/46,000 (85.3%)	40,453/45,601 (88.7%)	1		1		1	
CP ≥ 3	6787/46,000 (14.8%)	5148/45,601 (11.3%)	1.36 (1.29–1.43)	<0.001*	1.35 (1.29–1.42)	<0.001*	1.35 (1.28–1.42)	<0.001*
Total cholesterol ≥ 200mg/dL (n = 67,393)								
CP < 3	28,775/33,497 (85.9%)	29,966/33,896 (88.4%)	1		1		1	
CP ≥ 3	4722/33,497 (14.1%)	3930/33,896 (11.6%)	1.27 (1.20–1.34)	<0.001*	1.26 (1.19–1.34)	<0.001*	1.27 (1.20–1.35)	<0.001*
CCI scores = 0 (n = 92,661)								
CP < 3	37,382/44,090 (84.8%)	42,695/48,571 (87.9%)	1		1		1	
CP ≥ 3	6708/44,090 (15.2%)	5876/48,571 (12.1%)	1.30 (1.24–1.37)	<0.001*	1.30 (1.24–1.37)	<0.001*	1.30 (1.24–1.37)	<0.001*
CCI score = 1 (n = 25,231)								
CP < 3	11,672/13,656 (85.5%)	10,245/11,575 (88.5%)	1		1		1	
CP ≥ 3	1984/13,656 (14.5%)	1330/11,575 (11.5%)	1.29 (1.17–1.42)	<0.001*	1.28 (1.16–1.41)	<0.001*	1.26 (1.14–1.39)	<0.001*
CCI score ≥ 2 (n = 41,102)								
CP < 3	18,934/21,751 (87.1%)	17,479/19,351 (90.3%)	1		1		1	
CP ≥ 3	2817/21,751 (13.0%)	1872/19,351 (9.7%)	1.48 (1.36–1.61)	<0.001*	1.47 (1.35–1.60)	<0.001*	1.45 (1.33–1.58)	<0.001*

Abbreviations: SBP, Systolic blood pressure; DBP, Diastolic blood pressure; CCI, Charlson Comorbidity Index; * Conditional or unconditional logistic regression analysis, Significance at $P < 0.05$. † Stratified model for age, sex, income, and region of residence. ‡ Model 1 was adjusted for smoking, alcohol consumption, obesity and CCI scores. § Model 2 was adjusted for model 1 plus total cholesterol, SBP, DBP, and fasting blood glucose.

Supplementary Table S3. Crude and adjusted odd ratios of CP for BPH when participants are diagnosed with CP ≥ 1 within 2 years before index date.

Characteristics	N of BPH	N of Control	Odd ratios for BPH (95% confidence interval)					
	(exposure/total, %)	(exposure/total, %)	Crude†	*P*-value	Model 1†‡	*P*-value	Model 2†§	*P*-value
Total (n = 158,994)								
CP < 1	46,528/79,497 (58.5%)	51,928/79,497 (65.3%)	1		1		1	
CP ≥ 1	32,969/79,497 (41.5%)	27,569/79,497 (34.7%)	1.34 (1.31–1.37)	<0.001*	1.35 (1.32–1.37)	<0.001*	1.34 (1.31–1.37)	<0.001*
Age < 60 years old (n = 72,854)								
CP < 1	21,874/36,427 (60.1%)	24,074/36,427 (66.1%)	1		1		1	
CP ≥ 1	14,553/36,427 (40.0%)	12,353/36,427 (33.9%)	1.30 (1.26–1.34)	<0.001*	1.30 (1.26–1.34)	<0.001*	1.32 (1.28–1.36)	<0.001*
Age ≥ 60 years old (n = 86,140)								
CP < 1	24,654/43,070 (57.2%)	27,854/43,070 (64.7%)	1		1		1	
CP ≥ 1	18,416/43,070 (42.8%)	15,216/43,070 (35.3%)	1.37 (1.33–1.41)	<0.001*	1.36 (1.33–1.40)	<0.001*	1.35 (1.32–1.39)	<0.001*
Men (n = 158,994)								
CP < 1	46,528/79,497 (58.5%)	51,928/79,497 (65.3%)	1		1		1	
CP ≥ 1	32,969/79,497 (41.5%)	27,569/79,497 (34.7%)	1.33 (1.31–1.36)	<0.001*	1.33 (1.30–1.36)	<0.001*	1.33 (1.31–1.36)	<0.001*
Low income (n = 65,392)								
CP < 1	19,307/32,696 (59.1%)	22,067/32,696 (67.5%)	1		1		1	
CP ≥ 1	13,389/32,696 (41.0%)	10,629/32,696 (32.5%)	1.44 (1.39–1.49)	<0.001*	1.43 (1.39–1.48)	<0.001*	1.43 (1.38–1.47)	<0.001*
High income (n = 93,602)								
CP < 1	27,221/46,801 (58.2%)	29,861/46,801 (63.8%)	1		1		1	
CP ≥ 1	19,580/46,801 (41.8%)	16,940/46,801 (36.2%)	1.27 (1.23–1.30)	<0.001*	1.27 (1.23–1.30)	<0.001*	1.27 (1.24-1.31)	<0.001*
Urban residents (n = 72,576)								
CP < 1	20,683/36,288 (57.0%)	23,224/36,288 (64.0%)	1		1		1	
CP ≥ 1	15,605/36,288 (43.0%)	13,064/36,288 (36.0%)	1.34 (1.30–1.38)	<0.001*	1.33 (1.29–1.37)	<0.001*	1.34 (1.30–1.38)	<0.001*
Rural residents (n = 86,418)								
CP < 1	25,845/43,209 (59.8%)	28,704/43,209 (66.4%)	1		1		1	
CP ≥ 1	17,364/43,209 (40.2%)	14,505/43,209 (33.6%)	1.33 (1.29–1.37)	<0.001*	1.33 (1.29–1.36)	<0.001*	1.32 (1.29–1.36)	<0.001*
Underweight (n = 3790)								
CP < 1	986/1511 (65.3%)	1657/2279 (72.7%)	1		1		1	
CP ≥ 1	525/1511 (34.8%)	622/2279 (27.3%)	1.42 (1.23–1.63)	<0.001*	1.41 (1.22–1.63)	<0.001*	1.40 (1.22–1.62)	<0.001*
Normal weight (n = 52,793)								
CP < 1	15,095/24,946 (60.5%)	18,854/27,847 (67.7%)	1		1		1	
CP ≥ 1	9851/24,946 (39.5%)	8993/27,847 (32.3%)	1.37 (1.32–1.42)	<0.001*	1.36 (1.31–1.41)	<0.001*	1.37 (1.32–1.42)	0.001*
Overweight (n = 45,913)								
CP < 1	13,647/23,651 (57.7%)	14,344/22,262 (64.4%)	1		1		1	
CP ≥ 1	10,004/23,651 (42.3%)	7,918/22,262 (35.6%)	1.33 (1.28–1.38)	<0.001*	1.32 (1.27–1.37)	<0.001*	1.34 (1.29–1.39)	<0.001*
Obese (n = 56,498)								
CP < 1	16,800/29,389 (57.2%)	17,073/27,109 (63.0%)	1		1		1	
CP ≥ 1	12,589/29,389 (42.8%)	10,036/27,109 (37.0%)	1.27 (1.23–1.32)	<0.001*	1.26 (1.22–1.31)	<0.001*	1.29 (1.24–1.33)	<0.001*
Non-smoker (n = 68,807)								
CP < 1	22,524/36,299 (62.1%)	22,115/32,508 (68.0%)	1		1		1	
CP ≥ 1	13,775/36,299 (38.0%)	10,393/32,508 (32.0%)	1.30 (1.26–1.34)	<0.001*	1.30 (1.26–1.34)	<0.001*	1.31 (1.27–1.35)	<0.001*
Past and current smoker (n = 90,187)								
CP < 1	24,004/43,198 (55.6%)	29,813/46,989 (63.5%)	1		1		1	
CP ≥ 1	19,194/43,198 (44.4%)	17,176/46,989 (36.6%)	1.39 (1.35–1.43)	<0.001*	1.38 (1.34–1.42)	<0.001*	1.35 (1.32–1.39)	<0.001*
Alcohol consumption < 1 time a week (n = 74,667)								
CP < 1	24,023/39,014 (61.6%)	24,281/35,653 (68.1%)	1		1		1	
CP ≥ 1	14,991/39,014 (38.4%)	11,372/35,653 (31.9%)	1.33 (1.29–1.37)	<0.001*	1.33 (1.29–1.37)	<0.001*	1.32 (1.28–1.37)	<0.001*
Alcohol consumption ≥ 1 time a week (n = 84,327)								
CP < 1	22,505/40,483 (55.6%)	27,647/43,844 (63.1%)	1		1		1	

CP ≥ 1	17,978/40,483 (44.4%)	16,197/43,844 (36.9%)	1.36 (1.33–1.40)	<0.001*	1.35 (1.32–1.39)	<0.001*	1.34 (1.30–1.38)	<0.001*
SBP < 120 mmHg and DBP < 80 mmHg (n = 36,504)								
CP < 1	10,644/18,789 (56.7%)	11,178/17,715 (63.1%)	1		1		1	
CP ≥ 1	8145/18,789 (43.4%)	6537/17,715 (36.9%)	1.31 (1.25–1.36)	<0.001*	1.31 (1.26–1.37)	<0.001*	1.32 (1.26–1.38)	<0.001*
SBP ≥ 120 mmHg or DBP ≥ 80 mmHg (n = 122,490)								
CP < 1	35,884/60,708 (59.1%)	40,750/61,782 (66.0%)	1		1		1	
CP ≥ 1	24,824/60,708 (40.9%)	21,032/61,782 (34.0%)	1.34 (1.31–1.37)	<0.001*	1.33 (1.30–1.37)	<0.001*	1.34 (1.31–1.37)	<0.001*
Fasting blood glucose < 100 mg/dL (n = 89,618)								
CP < 1	27,353/45,407 (60.2%)	29,386/44,211 (66.5%)	1		1		1	
CP ≥ 1	18,054/45,407 (39.8%)	14,825/44,211 (33.5%)	1.31 (1.27–1.34)	<0.001*	1.30 (1.27–1.34)	<0.001*	1.31 (1.27–1.34)	<0.001*
Fasting blood glucose ≥ 100 mg/dL (n = 69,376)								
CP < 1	19,175/34,090 (56.3%)	22,542/35,286 (63.9%)	1		1		1	
CP ≥ 1	14,915/34,090 (43.8%)	12,744/35,286 (36.1%)	1.38 (1.33–1.42)	<0.001*	1.36 (1.32–1.41)	<0.001*	1.36 (1.32–1.41)	<0.001*
Total cholesterol < 200mg/dL (n = 91,601)								
CP < 1	26,808/46,000 (58.3%)	29,913/45,601 (65.6%)	1		1		1	
CP ≥ 1	19,192/46,000 (41.7%)	15,688/45,601 (34.4%)	1.37 (1.33–1.40)	<0.001*	1.36 (1.32–1.40)	<0.001*	1.35 (1.32–1.39)	<0.001*
Total cholesterol ≥ 200mg/dL (n = 67,393)								
CP < 1	19,720/33,497 (58.9%)	22,015/33,896 (65.0%)	1		1		1	
CP ≥ 1	13,777/33,497 (41.1%)	11,881/33,896 (35.1%)	1.29 (1.25–1.34)	<0.001*	1.29 (1.25–1.33)	<0.001*	1.30 (1.26–1.34)	<0.001*
CCI scores = 0 (n = 92,661)								
CP < 1	25,309/44,090 (57.4%)	31,185/48,571 (64.2%)	1		1		1	
CP ≥ 1	18,781/44,090 (42.6%)	17,386/48,571 (35.8%)	1.33 (1.30–1.37)	<0.001*	1.33 (1.30–1.37)	<0.001*	1.33 (1.30–1.37)	<0.001*
CCI score = 1 (n = 25,231)								
CP < 1	8006/13,656 (58.6%)	7502/11,575 (64.8%)	1		1		1	
CP ≥ 1	5650/13,656 (41.4%)	4073/11,575 (35.2%)	1.30 (1.24–1.37)	<0.001*	1.29 (1.23–1.36)	<0.001*	1.28 (1.22–1.35)	<0.001*
CCI score ≥ 2 (n = 41,102)								
CP < 1	13,213/21,751 (60.8%)	13,241/19,351 (68.4%)	1		1		1	
CP ≥ 1	8538/21,751 (39.3%)	6110/19,351 (31.6%)	1.40 (1.34–1.46)	<0.001*	1.39 (1.34–1.45)	<0.001*	1.37 (1.32–1.43)	<0.001*

Abbreviations: SBP, Systolic blood pressure; DBP, Diastolic blood pressure; CCI, Charlson Comorbidity Index; * Conditional or unconditional logistic regression analysis, Significance at $P < 0.05$. † Stratified model for age, sex, income, and region of residence. ‡ Model 1 was adjusted for smoking, alcohol consumption, obesity and CCI scores. § Model 2 was adjusted for model 1 plus total cholesterol, SBP, DBP, and fasting blood glucose.
